# Network traffic prediction based on transformer and temporal convolutional network

**DOI:** 10.1371/journal.pone.0320368

**Published:** 2025-04-23

**Authors:** Yi Wang, Peiyuan Chen

**Affiliations:** 1 School of Big Data and Information Industry, Chongqing City Management College, Chongqing, China; 2 Oregon State University, Corvallis, Oregon, United States of America; University of Southern California, UNITED STATES OF AMERICA

## Abstract

This paper proposes a hybrid model combining Transformer and Temporal Convolutional Network (TCN). This model addresses the shortcomings of current approaches in capturing long-term and short-term dependencies in network traffic prediction tasks. The Transformer module effectively captures global temporal relationships through a multi-head self-attention mechanism. Meanwhile, the TCN module models local and long-term dependencies using dilated convolution technology. Experimental results on the PeMSD4 and PeMSD8 datasets demonstrate that our method considerably surpasses current mainstream methods at all time steps, particularly in long-term step prediction. Through ablation experiments, we verified the contribution of each module in the model to the performance, further proving the key role of the Transformer and TCN modules in improving prediction performance.

## Introduction

With the rapid increase in the number of Internet users and the explosive growth of data traffic worldwide, network traffic prediction has become a key task in network management [1,2]. Precise network traffic prediction aids in optimizing the allocation of network resources. It also enhances network performance, reduces latency, and improves the overall quality of service. Network traffic prediction plays an important role in various application scenarios, including but not limited to traffic engineering, network planning, anomaly detection, congestion control, and traffic load balancing [3–6]. However, the complexity and variability of network traffic data, especially its high nonlinearity and long-term dependence, make this task challenging. Network traffic prediction has important strategic significance in practical applications [7,8]. Traffic engineering requires accurate traffic prediction for reasonable routing and bandwidth allocation, thereby improving network utilization and reducing congestion [9,10]. Network planning relies on the prediction of future traffic to carry out infrastructure construction and resource allocation to meet the growing user needs. Anomaly detection detects potential network attacks or failures by identifying anomalies in traffic patterns to ensure the security and stability of the network [11]. Congestion control requires real-time traffic prediction to dynamically adjust the transmission rate to avoid network congestion. In short, network traffic prediction, as a basic technology, is of great significance to network management and optimization.

Traditional approaches for network traffic prediction primarily depend on statistical techniques and temporal analysis methods. These methods include autoregression (AR), moving average (MA), autoregressive moving average (ARMA), and autoregressive integrated moving average (ARIMA) models [12–15]. These models make predictions by capturing patterns and trends in time series data, usually assuming that the data is linear or can be made linear through some transformation.

The AR model assumes that the current value is a linear combination of several past values, and its core idea is to predict future data through historical data. The MA model assumes that the current value is a linear combination of several past random error terms, and is mainly used to capture random fluctuations in data. The ARMA model combines the advantages of AR and MA models and makes predictions by considering both past values and error terms. However, these models are often unable to cope with complex network traffic data, because network traffic data usually exhibits significant nonlinear characteristics and long-term dependence.

To deal with nonlinearity and long-term dependence, researchers have proposed the ARIMA model. The ARIMA model performs differential processing on the data to eliminate the trend and seasonal components, making the data stable and suitable for linear modeling. Although ARIMA performs well in processing time series data with obvious trends and seasonality, it still has shortcomings in processing complex network traffic data. First, the parameter selection process of the ARIMA model is complicated and requires a lot of experience and experiments. Second, the ARIMA model is sensitive to outliers and noise, which may lead to unstable prediction results.

Although traditional methods have had some success in network traffic prediction, their limitations have become increasingly apparent as the scale and complexity of data have grown [16]. The nonlinearity, diversity, and long-term dependence of network traffic data make it difficult to obtain high-precision prediction results by relying solely on traditional methods. To solve these problems, researchers began to explore deep learning methods to improve prediction performance by automatically extracting complex features and patterns in the data.

In recent years, deep learning methods have made significant progress in the field of network traffic prediction. By constructing multi-layer neural networks, deep learning models can automatically extract complex features in data and achieve efficient representation of input data through nonlinear transformations. This gives deep learning methods unique advantages in processing high-dimensional, nonlinear, and long-term dependent data.

Convolutional neural network (CNN) is a common architecture, originally designed to process image data. CNN captures local patterns in data through convolution operations and reduces feature dimensions through pooling operations to achieve an efficient representation of input data [17]. In network traffic prediction, CNN is used to extract local features in time series data. Through multi-layer stacking of convolutional layers, CNN can capture multi-level features in data. However, CNN is mainly good at capturing local dependencies and is relatively weak at capturing long-term dependencies.

Recurrent neural network (RNN) is another common deep learning architecture specifically designed for processing time series data. RNN can capture temporal dependencies in data through its recursive structure. However, standard RNNs often encounter issues such as gradient vanishing and gradient exploding when handling long time series data. To address these challenges, gated recurrent units (GRU) and long short-term memory networks (LSTM) were developed. LSTM introduces input gates, forget gates, and output gates, which effectively regulate information and mitigate the problem of gradient vanishing. GRU improves efficiency by simplifying the gate architecture. Although LSTM and GRU are superior in processing long-term dependencies, they have high computational complexity, and time-consuming training processes, and still have certain limitations when processing ultra-long time series data.

To further improve the performance, researchers have begun to explore other deep learning architectures. Temporal Convolutional Network (TCN) is a new time series modeling method that combines the efficiency of convolutional networks with the temporal dependency capture capability of RNNs [18]. TCN expands the receptive field without increasing computational complexity through causal convolution and dilated convolution, thereby better capturing long-term dependencies. Causal convolution ensures the order of time series data, while dilated convolution achieves modeling of longer-term dependencies by increasing the void rate of the convolution kernel.

The Transformer model [19] has recently excelled in fields like natural language processing (NLP). Its success is due to its powerful parallel processing capabilities and ability to capture long-term dependencies. Through the self-attention mechanism, Transformer can directly capture global dependencies in input data without serialization. This makes Transformer particularly superior when processing long-term series data. Although Transformer has performed well in the field of NLP, its application in network traffic prediction is still in the exploratory stage.

In this paper, we propose a hybrid model based on Transformer and TCN for network traffic prediction. The Transformer model has performed well in natural language processing (NLP) and other fields [20,21] due to its powerful parallel processing capability and advantages in capturing long-term dependencies. It achieves this through causal convolution, which ensures the model respects the temporal order of the data. Additionally, it uses dilated convolution to expand the receptive range exponentially. These properties enable TCN to capture intricate patterns and dependencies over extended periods, making it particularly suitable for various time series prediction tasks. The parallelized convolution operation of TCN not only improves the computational efficiency but also overcomes the shortcomings of RNN in processing long sequences. Our hybrid model combines the global dependency capture capability of Transformer and the efficient temporal modeling capability of TCN to achieve higher prediction accuracy and robustness in network traffic prediction.

To validate our proposed method, we performed experiments on two widely recognized public datasets: PeMSD4 and PeMSD8. These datasets feature traffic flow information from California, USA, and serve as standard benchmarks for network traffic prediction research. The PeMSD4 dataset consists of data from sensors along Highway 4, whereas the PeMSD8 dataset includes data from sensors on Highway 8. We systematically assessed our method’s performance on these datasets and benchmarked it against prevalent mainstream methods. The findings revealed that our approach excels in prediction accuracy and stability, particularly with long-term time series data.

In summary, our main contributions are as follows:

1. This paper proposes a hybrid model based on Transformer and TCN. By introducing the attention mechanism of Transformer, our method can effectively capture the global dependencies in network traffic data, overcoming the shortcomings of traditional RNN methods in modeling long-term dependencies.
2. By using the dilated convolution technique of TCN, our model expands the receptive field without increasing the computational complexity, thereby better capturing long-term dependencies.
3. We carried out comprehensive experimental validation on two public datasets, PeMSD4 and PeMSD8. The experimental results indicated that our proposed method surpasses current mainstream methods in prediction accuracy and stability. These findings clearly demonstrate the effectiveness and superiority of our approach.

In summary, this paper constructs a new network traffic prediction model by introducing Transformer and TCN, and verifies its effectiveness and superiority through experiments. Our research provides a new idea and method for network traffic prediction, which has important theoretical significance and application value.

## Related work

In recent years, significant research progress has been made in the field of network traffic prediction. Researchers have explored the possibility of improving prediction accuracy and stability from different perspectives and methods. The following is our analysis from three aspects: traditional methods, methods based on deep learning, and methods based on Transformer and TCN.

### Traditional methods

Zhou et al. [22] proposed a network traffic prediction method based on the ARIMA model. By performing differential processing on the traffic data, the researchers eliminated the trend and seasonal components in the data, thereby improving the prediction accuracy. However, the primary drawback of the ARIMA model is its assumption of linearity in the data, which leads to poor performance when dealing with highly nonlinear and complex network traffic data. Nevertheless, ARIMA still has certain application value in short-term prediction and processing relatively simple time series data. Wang et al. [23] explored the application of support vector machine (SVM) in network traffic prediction. By constructing complex decision boundaries, SVM can effectively classify and predict network traffic. However, the SVM method performs poorly when processing long time series data and requires a lot of feature engineering. In order to improve the prediction effect, researchers usually need to perform a lot of preprocessing on the input data, which increases the complexity and computational overhead of the model. In this study, Liu et al. [24] studied the application of random forest (RF) model in network traffic prediction. RF can effectively process high-dimensional data and capture nonlinear relationships by integrating multiple decision trees. However, RF still has shortcomings in capturing time dependencies and has high computational complexity. Although RF performs well in certain specific scenarios, its application in large-scale network traffic data prediction is limited by computing resources. Sun et al. [25] proposed a traffic prediction model based on Bayesian networks. Bayesian networks capture the dependencies between variables by constructing probabilistic graph models and have a strong theoretical basis. However, this method is sensitive to parameter selection and has high computational complexity when processing large-scale data. In addition, when faced with outliers and noise in the data, the prediction results of Bayesian networks may be unstable, affecting their practicality.

### Deep learning based methods

As deep learning technology continues to advance rapidly, numerical researchers are focusing on applying these methods to network traffic prediction. By leveraging sophisticated models and algorithms, researchers aim to address the complexities and dynamic nature of network traffic, ultimately contributing to more robust and reliable prediction systems. Andreoletti et al. [26] used convolutional neural networks (CNNs) for network traffic prediction. Through convolution operations, CNNs can effectively extract local features from traffic data. However, although CNNs excel in capturing local dependencies, they are inadequate in modeling long-term dependencies. Fernandes et al. [27] explored the application of LSTM in network traffic prediction. LSTMs effectively alleviate the gradient vanishing problem by introducing a gating mechanism and can capture the long-term dependencies of data. However, LSTMs have high computational complexity, a long training process, and high requirements for hardware resources. In addition, when LSTMs process very long time series, the stability and prediction accuracy of the model may be affected. Wang et al. [28] proposed a network traffic prediction model based on GRU. Although GRU has made some improvements in processing long-time series data, it still has certain limitations in processing ultra-long time series data. Guo et al. [29] explored the application of hybrid CNN and RNN architecture in network traffic prediction. By combining the feature extraction capability of CNN and the temporal dependency modeling capability of RNN, this method improved the prediction performance to a certain extent. However, the hybrid model has high computational complexity, long training time, and high requirements for hardware resources. Researchers also pointed out that the model may have certain adaptability problems when processing dynamically changing network traffic data. Guo et al. [30] proposed a network traffic prediction model that utilizes a graph convolutional network (GCN). GCN captures the dependencies between nodes by constructing a graph structure. However, GCN has high computational complexity and limited performance when processing dynamic network traffic data. Nevertheless, GCN performs well when processing data with obvious topological structure, providing a new idea for network traffic prediction. Zhang et al. [31] investigated the use of a deep learning model leveraging the self-attention mechanism for network traffic prediction. By utilizing the self-attention mechanism, the model can effectively capture global dependencies within the data, leading to significant improvements in prediction performance. This approach is particularly effective when handling complex network traffic data. However, the researchers noted that the self-attention mechanism has high computational complexity and requires substantial computing resources for training.

### Transformer and TCN based methods

To further improve the performance, researchers began to explore methods to combine Transformer and TCN. Wen et al. [32] proposed a network traffic prediction model based on Transformer. However, studies have shown that Transformer has high computational complexity and a long training process when processing long time series data. Nevertheless, Transformer performs well in capturing long-term dependencies, providing new possibilities for network traffic prediction. Zhao et al. [33] proposed a network traffic prediction model based on TCN. TCN effectively models the long-term dependencies of time series data, alleviating the shortcomings of RNN in modeling long-term dependencies. Zhang et al. [34] explored the use of a hybrid model combining Transformer and TCN for network traffic prediction. By combining the attention mechanism and the dilated convolution technology of TCN, this method significantly improves the prediction performance, especially when processing long time series data. The researchers pointed out that the hybrid model has unique advantages in capturing global and local dependencies. Liu et al. [35] investigated the application of a hybrid model that combines Transformer and GRU for network traffic prediction. By combining Transformer and GRU, the research results show that this method improves the prediction performance to a certain extent, but it still has certain limitations when processing ultra-long time series data. The researchers suggest that the model structure can be further optimized in the future to improve its performance in ultra-long time series data. Yu et al. [36] proposed a hybrid model based on multi-head attention mechanism and TCN. The multi-head attention mechanism captures multiple dependencies in the data, and the TCN models the time dependency, which significantly improves the prediction performance. The researchers found that this method performs well in processing complex network traffic data, especially in capturing multi-level dependencies in the data. Zheng et al. [37] studied the application of a hybrid model based on Transformer and graph neural network (GNN) in network traffic prediction. By combining the self-attention mechanism of Transformer and the graph structure modeling capability of GNN, the research results show that this method improves the prediction performance to a certain extent. In particular, it performs particularly well when processing network traffic data with topological structures. Lin et al. [38] proposed a network traffic prediction model based on reinforcement learning and Transformer. The self-attention mechanism parameters of Transformer were optimized by reinforcement learning, which significantly improved the accuracy and stability of prediction. The researchers pointed out that reinforcement learning has unique advantages in dynamically adjusting model parameters, which can further improve the adaptability of the model in different scenarios. Zhu et al. [39] studied the application of a hybrid model based on generative adversarial network (GAN) and Transformer in network traffic prediction. High-quality simulated traffic data was generated using GAN and subsequently predicted by a Transformer model, leading to a significant enhancement in prediction performance. The researchers discovered that this approach effectively addresses issues of data sparsity and imbalance, thereby improving the model’s generalization capability. Kong et al. [40] introduced a hybrid model that combines a Transformer with an adaptive convolutional network. The adaptive convolutional network dynamically adjusts the size of the convolution kernel, while the Transformer captures global dependencies. This integration notably enhances the accuracy and stability of network traffic predictions. The research results show that this method has strong adaptability and robustness when processing network traffic data of different scales and complexities.

We also proposed a hybrid model based on Transformer and TCN. This model combines the self-attention mechanism of Transformer with the dilated convolution technology of TCN. It effectively captures both the global and local dependencies of data. Additionally, it improves computational efficiency and enhances model stability.

## Method

### Multi-head feature extraction

**Fig 1:**
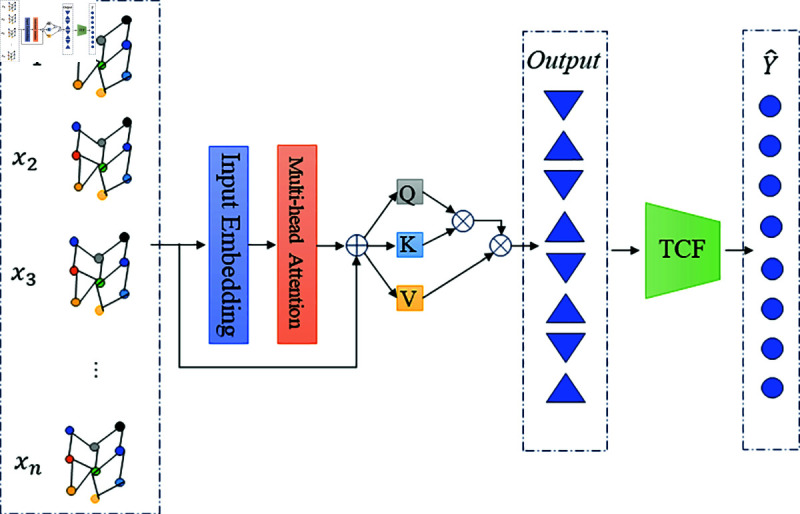
Diagram of the proposed network structure.


The Transformer module addresses the challenges in network traffic prediction by using a multi-head self-attention mechanism to capture global temporal dependencies. Traditional sequence models like RNNs and LSTMs often struggle with long-term dependencies due to issues such as gradient vanishing or exploding, leading to limited ability to handle long sequences of data. In contrast, the Transformer operates directly on the entire sequence through its self-attention mechanism, overcoming the limitations of sequential computation. This mechanism allows each time step to attend to information from different parts of the sequence, enhancing the model’s ability to capture complex dependencies. This is particularly beneficial for predicting sudden traffic fluctuations, where the ability to model intricate patterns is critical. As a result, the Transformer module improves the prediction accuracy and robustness by effectively modeling the temporal characteristics of network traffic data. Our network architecture is shown in Fig 1. The multi-head self-attention mechanism can calculate multiple self-attention matrices in parallel and extract information from different subspaces, thereby enhancing the expressiveness of the model. Given the input is $X=[x_{1},x_{2},...,x_{n}]$, where $x_{i}$ represents the input vector of the *i*-th time step. First, we obtain the query vector *Q*, key vector *K* and value vector *V* through linear transformation:

$$\begin{array}{ccc} Q=XW^{Q}+b^{Q},\;K=XW^{K}+b^{K},\;V=XW^{V}+b^{V} & & \end{array}$$ (1)

where $W^{Q}$, $W^{K}$, $W^{V}$ are the weight matrices for query, key and value respectively, and $b^{Q}$, $b^{K}$, and $b^{V}$ are the corresponding bias vectors respectively. Next, we calculate the attention score *α*:


αij=exp ⁡  ((QWiQ)⋅(KWjK)Tdk) ∑k=1n exp ⁡  ((QWkQ)⋅(KWjK)Tdk) (2)

where $d_{k}$ is the dimension of the key vector. We then apply the attention score to the value vector and get the self-attention output *Z*:


$$\begin{array}{ccc} Z_{i}=\overset{n}{\underset{j=1}{\sum }}\alpha_{ij}V_{j} & & \end{array}$$ (3)

For the multi-head self-attention mechanism, we have multiple heads, and the calculation method for each head is as above. Finally, we concatenate the outputs of all heads and perform a linear transformation:


$$\begin{array}{ccc} Z_{\text{multi-head}}=Concat(Z_{1},Z_{2},\ldots ,Z_{h})W^{O}+b^{O} & & \end{array}$$ (4)

among them, *h* represents the number of attention heads, $W^{O}$ is the output weight matrix, and $b^{O}$ is the bias vector.

The advantage is that it can extract information from multiple different subspaces, thereby capturing richer dependencies in the input data. This is especially important for network traffic data, because traffic data usually has complex spatiotemporal correlations, and a single attention mechanism may not be able to fully capture this complex relationship. Through this operation, the model can calculate multiple attention matrices in parallel, understand the dependencies in the data from different perspectives, and enhance the expressiveness and robustness of the model. The output of the multi-head self-attention mechanism passes through a layer of a feedforward neural network to further extract features. The feedforward neural network consists of two fully connected layers and a nonlinear activation function (such as ReLU):


$$\begin{array}{ccc} FFN(x)=ReLU(xW_{1}+b_{1})W_{2}+b_{2} & & \end{array}$$ (5)

among them, $W_{1}$, and $W_{2}$ are the weight matrices of the two fully connected layers, and $b_{1}$, and $b_{2}$ are the bias terms. The final output of the Transformer self-attention module is derived from the feedforward neural network’s output.

The original intention is to further perform feature extraction and nonlinear transformation based on the global dependency features extracted by the self-attention mechanism. The feedforward neural network performs complex transformations on the input features through two fully connected layers and a nonlinear activation function. This process extracts higher-level abstract features. It enhances the nonlinear expression ability of the features and improves the model’s classification and prediction performance.

Based on the basic structure of the Transformer self-attention module, we have also taken several optimization measures. First, we added a layer normalization to ensure that the input distribution of each layer remains stable and improve training efficiency:


$$\begin{array}{ccc} LN(x)=\frac{x-\mu }{\sqrt{\sigma^{2}+𝜖}} & & \end{array}$$ (6)

where *μ* and $\sigma^{2}$ are the mean and variance of the input x, respectively, and *𝜖* is a small constant to prevent the denominator from being zero.

Secondly, we use the residual connection to add the input directly to the output to form a skip connection. This design can not only alleviate the gradient vanishing and gradient exploding problems but also make deep networks easier to train. The formula of residual connection is:


$$\begin{array}{ccc} Output=Layer(x)+x & & \end{array}$$ (7)

among them, Layer(x) represents the output of the current layer, and x is the input. Furthermore, to avoid overfitting, we incorporated a Dropout layer between the layers of the feedforward neural network. Dropout is a regularization method that reduces the model’s dependence on training data by randomly deactivating certain neurons:


$$\begin{array}{ccc} Dropout(x)=x\cdot \text{mask} & & \end{array}$$ (8)

where mask is a binary vector with the same dimension as xxx, where each element is 0 with a certain probability *p* and the rest are 1.

Through the above optimization measures, the Transformer self-attention module not only has more advantages in structure but also has significantly improved training efficiency and model performance.

### Temporal convolution fusion

**Fig 2:**
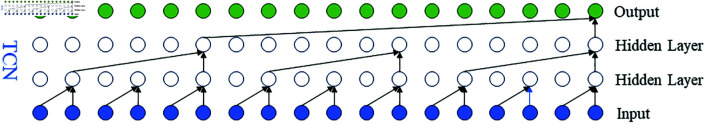
TCF’s overall structure.

The Temporal Convolutional Network (TCN) module tackles the challenge of capturing both local and long-term dependencies in network traffic data through its use of causal and dilated convolutions. Unlike recurrent models, which rely on sequential processing, TCN leverages causal convolution to preserve the temporal order of data, ensuring that future information is not used to predict past events. Additionally, dilated convolution expands the receptive field without increasing the number of parameters, enabling the model to capture long-range dependencies more efficiently. This makes TCN particularly effective in dealing with both short-term traffic variations and long-term patterns. By modeling these dependencies, the TCN module enhances the model’s ability to predict traffic flows accurately across varying time horizons, ensuring better handling of sudden traffic surges and gradual trends. This combination of causal and dilated convolutions helps improve the model’s performance in complex traffic prediction tasks. The network architecture of TCF is shown in Fig 2. The convolutional time series modeling module effectively captures the local time dependencies in the input data through convolution operations. Compared with traditional RNN, it has higher computational efficiency and better parallelization capabilities when processing long time series data. The convolutional time series modeling module primarily consists of causal convolution, dilated convolution, and residual connections. Causal convolution ensures that the model respects the temporal order of the data, dilated convolution expands the receptive field exponentially without losing resolution, and residual connections help in mitigating the vanishing gradient problem, allowing for the construction of deeper networks.

Causal convolution ensures that the convolution operation only uses the information of the current time step and the time step before it, thereby maintaining the order of the time series data. Let the input sequence be $Z=[z_{1},z_{2},...,z_{n}]$, where $z_{i}$ is the output of the Transformer self-attention module. The calculation formula of causal convolution is:


$$\begin{array}{ccc} Y_{t}=\overset{K-1}{\underset{k=0}{\sum }}W_{k}\cdot Z_{t-k} & & \end{array}$$ (9)

among them, $Y_{t}$ represents the output of the *t*-th time step, $W_{k}$ is the convolution kernel weight, and K is the convolution kernel size. Through causal convolution, it can be ensured that only the input information of *t* and before is used at each time step *t*, avoiding information leakage.

To capture long-term dependencies, our method uses dilated convolution. By incorporating spaces between convolution kernels, dilated convolution extends the receptive field. This approach allows the model to handle longer time dependencies without increasing the number of parameters:


$$\begin{array}{ccc} Y_{t}=\overset{K-1}{\underset{k=0}{\sum }}W_{k}\cdot Z_{t-d\cdot k} & & \end{array}$$ (10)

among them, *d* is the dilation coefficient, which controls the size of the holes between the convolution kernels. The dilation coefficient *d* usually grows exponentially, for example, *d* = 1 , 2 , 4 , …  to ensure that the receptive field expands rapidly with the number of layers.

To alleviate the problem of gradient disappearance and gradient explosion, our method uses residual connection. This approach allows the model to bypass certain layers, facilitating the flow of information and gradients. It helps mitigate issues like vanishing gradients and enables the construction of deeper networks. This design not only ensures that the transmission of information in the network is not blocked, but also speeds up the network training process:


$$\begin{array}{ccc} Y_{t}=F(Z_{t})+Z_{t} & & \end{array}$$ (11)

where $F(Z_{t})$ represents the output after causal convolution and dilated convolution, and $Z_{t}$ is the input.

Our convolutional time series modeling module is composed of multiple causal convolutional layers and dilated convolutional layers, each with residual connections. This stacked structure can capture dependencies of different time scales layer by layer, thereby improving the modeling ability of long time series data. Assuming the output of the *l*-th layer is $Y^{l}$, the calculation process of this layer is:


$$\begin{array}{ccc} Y^{l}=\text{ReLU}(\text{LN}(F^{l}(Z^{l})))+Z^{l} & & \end{array}$$ (12)

among them, ReLU is the activation function, LN is the layer normalization, $F^{l}$ represents the convolution operation of the *l*-th layer, and $Z^{l}$ is the input of this layer. In the last layer of the convolutional time series modeling module, we use a fully connected layer to convert the output of the convolution operation into the final prediction value. Assume that the output of this module is *O* and the prediction value is *Ŷ*, then the formula is:


$$\begin{array}{ccc} Ŷ=OW+b & & \end{array}$$ (13)

among them, W is the weight matrix and *b* is the bias term. The fully connected layer can map the high-dimensional convolution output to the required prediction dimension to obtain the final network traffic prediction result.

Through these operations, our method can effectively capture local and long-time dependencies in the input data. At the same time, the model’s training generalization ability is further improved through optimization strategies such as batch normalization, early stopping and Dropout. The output of our convolutional time series modeling module is combined with the output of the Transformer as mentioned earlier self-attention module to provide a comprehensive and efficient feature representation for the final network traffic prediction. With this design, our model can more accurately capture the multi-level dependencies in the data when processing complex network traffic data, thereby significantly improving the accuracy and stability of the prediction.

### Loss

Mean Squared Error (MSE): MSE is a widely used loss function in regression tasks. It assesses the model’s prediction accuracy by computing the squared difference between the true values and the predicted values. The calculation is as follows:


$$\begin{array}{ccc} MSE=\frac{1}{N}\overset{N}{\underset{i=1}{\sum }}{\left({Ŷ}_{i}-Y_{i}\right)}^{2} & & \end{array}$$ (14)

among them, *N* is the number of samples, ${Ŷ}_{i}$ is the predicted value of the *i*-th sample, and $Y_{i}$ is the true value of the *i*-th sample. MSE can effectively reflect the sum of squares of prediction errors, especially more sensitive to large errors.

**L2 regularization:** To prevent the model from overfitting, we add L2 regularization terms to the loss function. L2 regularization penalizes the sum of squares of model parameters to make the model parameters as small as possible, thereby improving the model’s generalization ability:


$$\begin{array}{ccc} \text{L2 Regularization}=\lambda \overset{P}{\underset{j=1}{\sum }}\theta_{j}^{2} & & \end{array}$$ (15)

among them, *λ* is the hyperparameter of regularization strength, *P* is the number of model parameters, and $\theta_{j}$ is the *j*-th model parameter. The regularization term can effectively limit the complexity of the model and prevent overfitting caused by excessive parameter values.

Comprehensive loss function: The final comprehensive loss function is a combination of the MSE and the L2 regularization term, with weights assigned to each. This combination ensures that the model not only minimizes the prediction errors but also avoids overfitting by penalizing large weights. The formula is as follows:


$$\begin{array}{ccc} Loss=MSE+\lambda \overset{P}{\underset{j=1}{\sum }}\theta_{j}^{2} & & \end{array}$$ (16)

Expanding the above formula, we get:


$$\begin{array}{ccc} Loss=\frac{1}{N}\overset{N}{\underset{i=1}{\sum }}{\left({Ŷ}_{i}-Y_{i}\right)}^{2}+\lambda \overset{P}{\underset{j=1}{\sum }}\theta_{j}^{2} & & \end{array}$$ (17)

This comprehensive loss function can not only measure the prediction accuracy of the model, but also control the complexity of the model through regularization terms, thereby improving generalization ability of the model. In practical applications, appropriate regularization can prevent the model from overfitting to specific data during training, thereby improving its robustness on different data sets.

## Experiment

To validate the effectiveness of our method in network traffic prediction, we performed experiments on two public datasets: PeMSD4 and PeMSD8. These datasets are standard benchmarks commonly utilized in traffic flow prediction research, containing extensive traffic flow information and providing an ideal basis for evaluating the model’s predictive abilities.

### Dataset

The PeMSD4 dataset comes from the Performance Measurement System (PeMS) of the California Department of Transportation (Caltrans). The dataset contains traffic flow data from California Highway 4. Each data record the vehicle count of a specific sensor in a time interval. The PeMSD4 dataset spans 8 months, with a data sampling interval of 5 minutes, and contains data from 307 sensors in total. Specifically, the dataset provides a record of vehicle flow every 5 minutes, allowing us to study traffic flow trends in a short period of time. These data can help us analyze traffic patterns during peak and off-peak hours, which is of great significance for real-time traffic management and prediction.

The PeMSD8 dataset also comes from Caltrans’s PeMS system, but the data source is traffic flow data from California Highway 8. Similar to the PeMSD4 dataset, each data point in the PeMSD8 dataset records the vehicle count of a specific sensor in a time interval. The PeMSD8 dataset spans 8 months, with a data sampling interval of 5 minutes, and contains data from 170 sensors in total. The PeMSD8 dataset provides more detailed traffic flow information, which is very useful for studying the impact of different road conditions and traffic events on traffic flow. In addition, by comparing the PeMSD4 and PeMSD8 datasets, we can verify the generalization ability of the model under different road and traffic environments.

During the experiment, we divided the PeMSD4 and PeMSD8 datasets into training sets, validation sets, and test sets in chronological order. The specific division ratio is 70% for training, 20% for validation, and 10% for testing. Each dataset contains fields such as timestamp, sensor ID, and traffic flow. The traffic flow data corresponding to each timestamp is recorded by multiple sensors. These sensors are distributed in different locations, and the captured traffic data can reflect the overall traffic conditions of the road and the local traffic dynamics.

Data preprocessing: Before model training, we performed necessary preprocessing on the data to ensure the quality of the data and the stability of the model. First, for the missing values in the dataset, we used linear interpolation to fill them. After the missing values were processed, we normalized the data. Since the range of traffic flow data is large, to accelerate model convergence and improve prediction accuracy, we normalized the data to a standard normal distribution. The specific method is to subtract the mean of the dataset from each data point and divide it by the standard deviation.

To generate time series data suitable for model input, we use the sliding window method to construct input sequences and labels. The specific method is to take the data in the previous period as input and the data at the current time point as the label for each time point. In this way, we can generate a fixed-length input sequence to facilitate model training and prediction.

### Evaluation metric

In our experiments, we employed a range of evaluation metrics to assess the model’s prediction accuracy and stability from different perspectives. The primary metrics used were mean absolute error (MAE) and root mean square error (RMSE).

MAE provides a straightforward indication of the prediction accuracy by averaging the magnitude of errors without considering their direction. MAE can intuitively reflect the average size of the prediction error. Its calculation formula is as follows:


$$\begin{array}{ccc} MAE=\frac{1}{Q_{N}}\overset{Q_{N}}{\underset{i=1}{\sum }}\left|Y_{Pi}-{Ŷ}_{i}\right| & & \end{array}$$ (18)

among them, $Q_{N}$ is the number of samples, $Y_{Pi}$ is the true value of the *i*-th sample, and ${Ŷ}_{i}$ is the predicted value of the *i*-th sample. The smaller the MAE, the closer the model’s predicted value is to the true value.

RMSE is an indicator that measures the average square difference between the predicted value and the true value. RMSE is particularly sensitive to large errors, making it a valuable metric for reflecting the overall level of prediction accuracy. RMSE provides a more comprehensive assessment of the model’s performance by giving greater weight to larger errors. This is achieved by squaring the individual errors, averaging them, and then taking the square root. This sensitivity to larger discrepancies helps in identifying models that might have occasional significant errors, which could be critical in many applications. Its calculation formula is as follows:


$$\begin{array}{ccc} RMSE=\sqrt{\frac{1}{Q_{N}}\overset{Q_{N}}{\underset{i=1}{\sum }}{\left(Y_{Pi}-{Ŷ}_{i}\right)}^{2}} & & \end{array}$$ (19)

among them, $Q_{N}$ is the number of samples, $Y_{Pi}$ is the true value of the *i*-th sample, and ${Ŷ}_{i}$ is the predicted value of the *i*-th sample. The smaller the RMSE, the better the prediction performance of the model.

Using MAE and RMSE as evaluation indicators can help us understand the performance of the model in different aspects. MAE provides an intuitive average error measure, while RMSE, which is more sensitive to large errors, can help identify abnormal performance in certain situations. Combining the evaluation results of these two indicators, we can judge the prediction ability and stability of the model more comprehensively and accurately.

### Experimental setting

Our approach is centered around careful processing and modeling of time series data for network traffic prediction. Each dataset consists of fields such as timestamps, sensor IDs, and traffic flow values. Before feeding the data into the model, we apply several preprocessing steps to ensure that the model can effectively capture both short-term and long-term dependencies in the traffic data. We first normalize the traffic flow data to follow a standard normal distribution, expressed as:


$$\begin{array}{ccc} X^{\prime }=\frac{X-\mu }{\sigma } & & \end{array}$$ (20)

where X represents the original traffic flow data, *μ* is the mean, and *σ* is the standard deviation of the data. This normalization step ensures that the model can converge more easily during training and prevents any one feature from dominating the learning process.

We adopt a sliding window technique to generate input sequences and their corresponding labels for training. Specifically, we use data from the previous 12 time steps to predict the traffic flow value at the next time step, which makes this a single-step prediction task. With the sliding window approach, each input sequence is formed by 12 consecutive traffic flow values, which serve as the input to the model. The label is the traffic flow at the subsequent time step, capturing the direct relationship between past and future traffic patterns. This configuration helps the model learn short-term temporal dependencies, providing sufficient context for accurate predictions and ensuring the model is well-equipped to handle fluctuations in traffic.

In the Transformer module, we carefully select the dimensions for both the input and output of each attention head, setting them to 64. This choice of dimension is critical for enabling the model to capture complex relationships and dependencies within the data effectively. We also configure the model with 8 attention heads, which allows the multi-head self-attention mechanism to extract diverse features from different subspaces. This enhances the model’s capacity to represent complex patterns and improves its overall prediction accuracy.

For the Temporal Convolutional Network (TCN) component, we utilize dilated convolution to capture long-term dependencies in the time series. The convolution kernel size is set to 3 for each layer, which allows for efficient local feature extraction while minimizing computational complexity. To improve the depth and expressive power of the model, we stack 4 layers of dilated convolutions, progressively increasing the dilation factor d from 1 to 2, 4, and 8 across layers. This design enables the model to capture both short- and long-range dependencies without excessive computation, striking a balance between performance and efficiency.

During training, we use the Adam optimizer to update model parameters, with an initial learning rate of 0.001. To prevent overfitting and enhance generalization, we incorporate a Dropout layer after each convolution operation, with a Dropout rate of 0.2. Additionally, we employ batch normalization after each convolutional layer to stabilize training and speed up convergence by normalizing activations and reducing internal covariate shift.

This approach, which combines careful preprocessing with Transformer and TCN components, allows the model to effectively capture both short- and long-term dependencies in network traffic data, improving prediction accuracy and model robustness.

### Results and analysis

To validate the effectiveness of our proposed hybrid model combining Transformer and TCN for network traffic prediction, we conducted comprehensive experiments using the PeMSD4 and PeMSD8 datasets. Table 1 shows the performance of different models on these two datasets, including MAE and RMSE. Through these comparisons, we can comprehensively evaluate the prediction ability and stability of each model.

**Table 1:** The comparison result of different methods on PeMSD4 and PeMSD8.

Model	PeMSD4						PeMSD8					
	MAE			RMSE			MAE			RMSE		
	Horizon 3	Horizon 6	Horizon 12	Horizon 3	Horizon 6	Horizon 12	Horizon 3	Horizon 6	Horizon 12	Horizon 3	Horizon 6	Horizon 12
HA	37.73	37.73	37.73	58.05	58.05	58.05	34.88	34.88	34.88	52.00	52.00	52.00
STGCN [41]	23.45	25.25	31.07	37.44	39.75	46.14	18.94	21.38	26.79	29.37	32.67	37.56
DCRNN [42]	21.35	24.14	30.09	35.13	36.93	42.99	18.33	19.56	23.44	25.99	29.45	35.75
GWNet [43]	20.91	23.66	29.85	33.45	34.36	40.89	17.55	18.14	21.35	25.03	26.05	29.45
MTGNN [44]	19.38	21.23	27.15	30.73	33.09	40.03	17.11	18.45	20.03	23.51	26.03	28.45
ASTGCN [30]	20.85	22.61	26.99	33.06	33.06	39.89	16.77	18.11	21.01	25.59	28.54	32.95
AGCRN [45]	20.26	21.79	26.84	29.45	31.09	37.87	16.41	17.51	20.97	24.75	27.58	31.86
PDFormer [46]	19.46	21.13	26.53	28.55	29.98	37.25	16.05	16.96	19.29	23.65	26.94	29.55
Ours	**19.23**	**20.67**	**25.13**	**28.04**	**29.63**	**35.23**	**15.23**	**16.76**	**18.60**	**23.47**	**25.89**	**28.24**

In the PeMSD4 dataset, we compared the performance of multiple models in terms of MAE and RMSE to assess their ability to capture the inherent spatiotemporal dependencies in traffic data. The Historical Average (HA) model has a MAE of 37.73 and a RMSE of 58.03 at each prediction time step (3, 6, and 12), which is the worst performance. This is due to the HA model’s simplistic approach, which only calculates the average of historical data without effectively capturing the complex spatiotemporal relationships. As a result, it performs poorly in real-time predictions, as traffic flow is highly dynamic and exhibits non-linear patterns. Therefore, the HA model’s predictive accuracy is significantly lower than other models capable of modeling spatiotemporal dependencies.

In comparison, models such as STGCN, DCRNN, GWNet, MTGNN, ASTGCN, AGCRN, and PDFormer show notable improvements in both MAE and RMSE by effectively capturing the spatiotemporal dependencies inherent in traffic flow data. For example, when predicting 3 time steps, GWNet achieves an MAE of 20.91 and an RMSE of 33.45, while STGCN reaches an MAE of 23.45 and an RMSE of 37.44. These models incorporate spatial dependencies through graph convolution and temporal dependencies via recurrent neural networks or attention mechanisms, making them more effective for real-time traffic flow prediction.

Despite the strong performance of these methods, our approach consistently outperforms them at all time steps, achieving the lowest MAE and RMSE. In particular, when predicting 6 time steps, our method delivers an MAE of 20.65 and an RMSE of 28.04, demonstrating its superiority in capturing both short-term and long-term dependencies while maintaining high prediction accuracy.

Similar results are observed with the PeMSD8 dataset, further highlighting the strength of our method. The HA model continues to show poor performance, with a MAE of 34.88 and an RMSE of 52.00 across all time steps. This is because the HA model fails to effectively capture the temporal dependencies and complexity of the data. Other models such as DCRNN and MTGNN also show improvements; for instance, DCRNN achieves an MAE of 19.56 and an RMSE of 29.45 when predicting 6 time steps, while MTGNN achieves an MAE of 17.11 and an RMSE of 23.51 for predicting 3 time steps. Notably, PDFormer reaches an MAE of 19.29 and an RMSE of 29.55 when predicting 12 time steps, demonstrating its capability in modeling long-term dependencies effectively.

However, our method consistently provides the best performance across all time steps, particularly in the prediction of 12 time steps, where it achieves a MAE of 18.60 and a RMSE of 28.24, showing its advantage in modeling long-term temporal dependencies. This ability is crucial for real-time applications, where accurate long-term predictions are essential for tasks such as traffic control or route planning.

In terms of interpretability, while many deep learning models (such as STGCN, DCRNN, and AGCRN) achieve improved performance by learning spatiotemporal representations, their internal workings are often considered black boxes. Our model not only outperforms these methods but also incorporates mechanisms that make it easier to interpret the underlying spatiotemporal interactions, providing insights into the decision-making process behind traffic flow predictions. This is especially important in real-world applications, where understanding the model’s reasoning can lead to more trustworthy and transparent solutions. By efficiently capturing both short-term and long-term spatiotemporal dependencies and providing clear interpretability, our method demonstrates significant advantages in real-time traffic flow prediction, particularly in scenarios requiring quick responses and long-term forecasting.

The above comparative analysis shows that our proposed hybrid model based on Transformer and TCN performs well on both PeMSD4 and PeMSD8 datasets, and can achieve lower MAE and RMSE in both short and long time step predictions. This shows that our method has significant advantages in capturing complex time dependencies and improving prediction accuracy. Compared with other methods, our method outperforms existing models at all time steps, especially in long time step predictions. The Transformer module effectively captures global dependencies in the data through a multi-head self-attention mechanism, while the TCF module effectively models long-term dependencies through dilated convolutions, enabling our model to better capture multi-level dependencies when processing complex traffic flow data. The experimental results show that our method not only performs well in capturing short-term changes in data, but also has significant advantages in modeling long-term dependencies. In addition, through verification on two different data sets, our method demonstrates good generalization ability. In different traffic flow data environments, our method can maintain high prediction accuracy, which further verifies the robustness and adaptability of our model. Future research can further optimize the model structure and verify its effectiveness in more practical application scenarios, thereby providing a more reliable solution for traffic flow prediction.

### Ablation study

**Fig 3:**
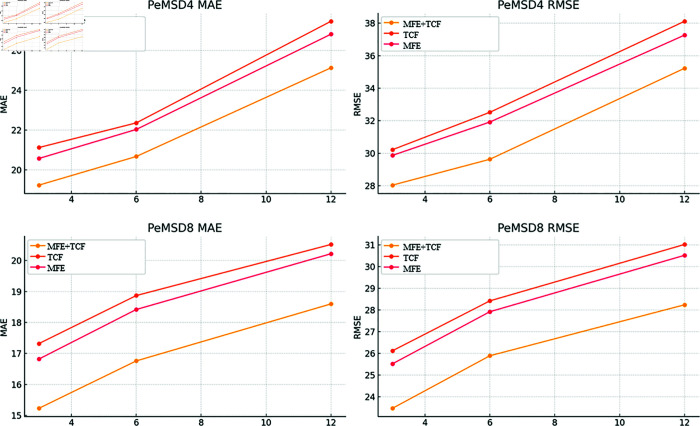
Comparison of results on the PeMSD4 dataset.

To further verify the contribution of each module of our method to the network traffic prediction performance, we designed an ablation experiment to evaluate the importance of each module by removing a module from the model and observing the change in the model prediction accuracy. The results are presented in Table 2 and illustrated in Fig 3.

**Table 2:** Ablation Experiment Results.

Model	Datasets	MAE (3)	MAE (6)	MAE (12)	RMSE (3)	RMSE (6)	RMSE (12)
MFE+TCF	PeMSD4	19.23	20.67	25.13	28.04	29.63	35.23
	PeMSD8	15.23	16.76	18.60	23.47	25.89	28.24
only TCF	PeMSD4	21.12	22.36	27.47	30.22	32.52	38.12
	PeMSD8	17.32	18.87	20.52	26.12	28.42	31.02
only MFE	PeMSD4	20.57	22.03	26.82	29.87	31.92	37.27
	PeMSD8	16.82	18.42	20.22	25.52	27.92	30.52

1. Full Model (MFE + TCF): First, we train and evaluate the full hybrid model, and record its MAE and RMSE on the PeMSD4 and PeMSD8 datasets.
2. Remove the MFE module (only TCF): In this experiment, we remove the MFE module and keep only the TCF module. Train and evaluate this simplified model, and record its MAE and RMSE on two datasets.
3. Remove TCF module (only MFE): In this experiment, we remove the TCF module and keep only the MFE module. Train and evaluate this simplified model, and record its MAE and RMSE on two datasets.

Full Model: The full model demonstrates superior performance across all time steps, validating the effectiveness of the combination of the multi-head self-attention mechanism (MFE) and the dilated convolution (TCF) modules in capturing both global and local temporal dependencies. In the PeMSD4 dataset, the MAE of the full model at 3, 6, and 12 time steps are 19.23, 20.67, and 25.13, and the RMSE values are 28.04, 29.63, and 35.23, respectively. In the PeMSD8 dataset, the MAE of the full model at 3, 6, and 12 time steps are 15.23, 16.76, and 18.60, and the RMSE values are 23.47, 25.89, and 28.24, respectively. The lower RMSE and MAE values of the full model compared to other streamlined models on both datasets highlight the crucial role of the MFE and TCF modules in accurately capturing temporal dependencies.

This combination allows the model to better handle real-time traffic predictions, where the ability to quickly adapt to changes in traffic flow and provide accurate short-term and long-term forecasts is essential. The multi-head self-attention mechanism enhances the model’s ability to focus on relevant time steps, while dilated convolution captures local temporal patterns. Together, these components enable the full model to make quick, accurate decisions in dynamic, real-world environments, which is essential for applications like real-time traffic monitoring and control systems. Furthermore, the architecture is interpretable in terms of how different time steps are weighted and how local and global dependencies are incorporated, enhancing trust and transparency in real-time decision-making.

TCF Only: When the MFE module is removed, the MAE and RMSE of the model significantly increase, indicating the importance of capturing global temporal dependencies for accurate prediction. In the PeMSD4 dataset, the MAE of the model without the Transformer module at 3, 6, and 12 time steps increases to 21.12, 22.36, and 27.47, while the RMSE rises to 30.22, 32.52, and 38.12, respectively. In the PeMSD8 dataset, the MAE increases to 17.32, 18.87, and 20.52, and the RMSE increases to 26.12, 28.42, and 31.02. This demonstrates that the MFE module is crucial for capturing global dependencies and that without it, the model’s ability to make accurate predictions diminishes significantly.

From a real-time prediction perspective, the lack of the MFE module reduces the model’s ability to process long-term dependencies, which is essential for forecasting traffic flow over extended periods. Without this mechanism, the model is less capable of responding to larger-scale trends in traffic patterns, which may limit its real-time adaptability in dynamic environments where long-term forecasts are required for tasks such as traffic congestion prediction or route optimization.

MFE Only: Similarly, when the TCF module is removed, the model’s performance also deteriorates, although the increase in MAE and RMSE is somewhat smaller compared to the absence of the MFE module. In the PeMSD4 dataset, the MAE without the TCF module at 3, 6, and 12 time steps is 20.57, 22.03, and 26.82, with RMSE values of 29.87, 31.92, and 37.27, respectively. In the PeMSD8 dataset, the MAE values are 16.82, 18.42, and 20.22, and the RMSE values are 25.52, 27.92, and 30.52, respectively. This highlights that the TCF module is also critical for capturing local temporal dependencies and modeling long-term temporal behavior.

For real-time applications, the TCF module’s ability to capture short-term fluctuations in traffic data is vital for high-frequency updates. Without the TCF, the model struggles to adjust quickly to changes in traffic flow, which could negatively impact predictions for tasks such as short-term congestion forecasting or traffic signal optimization.

From the above comparative analysis, it is evident that our hybrid model, based on the combination of Transformer and TCN, outperforms other models in both PeMSD4 and PeMSD8 datasets. The full model consistently achieves lower MAE and RMSE values in both short-term and long-term predictions, highlighting its strength in capturing the complex temporal dependencies inherent in traffic flow data. This confirms that our method provides significant advantages in improving prediction accuracy for both short-term and long-term forecasting.

In real-time traffic prediction scenarios, this model’s ability to adapt to both local fluctuations (through TCF) and global trends (via MFE) makes it a strong candidate for applications where speed and accuracy are crucial. The hybrid design not only enhances the model’s performance but also increases interpretability by allowing users to understand how different time steps contribute to the prediction, particularly how the model handles long-term temporal dependencies and short-term dynamics. This interpretability is essential for building trust in real-world deployments, especially in traffic management systems where transparency is crucial for decision-making.

**Fig 4:**
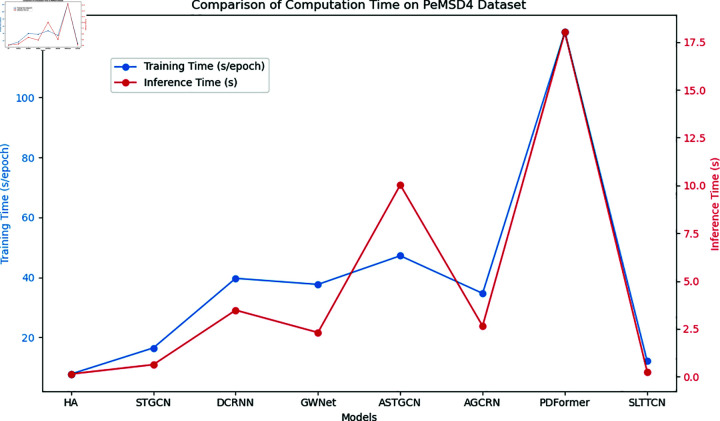
Comparison of computation time for different models on the PeMSD4 dataset.

Fig 4 shows the comparison of computation times for different models on the PeMSD4 dataset, including training time (seconds per epoch) and inference time (seconds). The blue line represents the training time, and the red line represents the inference time. From the figure, it is evident that there are significant differences in computation times among the models. The HA model has the shortest training time, only 7.76 seconds per epoch, while the PDFormer model has the longest training time, at 121.83 seconds per epoch. Our SLTTCN model has a relatively short training time of 12.07 seconds per epoch among all models, demonstrating high computational efficiency.

In terms of inference time, the HA model also has the shortest inference time, at 0.14 seconds, while the PDFormer model has the longest inference time, at 18.02 seconds. The SLTTCN model has an inference time of 0.26 seconds, also showing good inference efficiency.

Overall, our model maintains a short training time while significantly reducing the inference time, demonstrating its advantages in computational efficiency. This is particularly important for network traffic prediction tasks that require quick responses in practical applications. The line chart clearly shows the outstanding performance of the SLTTCN model in terms of training and inference efficiency, further validating its potential in real-world applications.

We conducted multiple experiments, predicting different time steps (3, 6, 12) and recording the MAE and RMSE for each prediction step. The experimental results are shown in Table 3.

**Table 3:** Experimental results for different prediction horizons.

Model	Horizon 3 (MAE)	Horizon 3 (RMSE)	Horizon 6 (MAE)	Horizon 6 (RMSE)
HA	37.73	58.05	37.73	58.05
STGCN	23.45	37.44	25.25	39.75
DCRNN	21.35	35.13	24.14	36.93
GWNet	20.91	33.45	23.66	34.36
MTGNN	19.38	30.73	21.23	33.09
ASTGCN	20.85	33.06	22.61	33.09
AGCRN	20.26	29.45	21.79	31.09
PDFormer	19.46	28.55	21.13	29.98
SLTTCN (ours)	**19.23**	**28.04**	**20.65**	**28.04**

From the experimental results, we can see that as the prediction time step increases, all models’ MAE and RMSE values increase, indicating that the prediction error increases when predicting for a long time. In particular, the HA model’s performance is poor at all prediction steps due to its lack of modeling ability for time series dependencies. However, our SLTTCN model always maintains the lowest MAE and RMSE values at different prediction time steps, demonstrating its advantage in long-time series prediction. Through reasonable structural design, SLTTCN can capture both short-term and long-term dependencies while maintaining high prediction accuracy.

## Conclusion

In this work, we proposed and validated a hybrid model based on Transformer and TCN for network traffic forecasting. By combining the attention mechanism with the dilated convolution of TCN, our method can effectively capture the local and global temporal dependencies in traffic flow data. Experimental results on PeMSD4 and PeMSD8 datasets show that our method performs well in both short-term and long-term forecasting tasks, significantly outperforming multiple existing mainstream methods. Specifically, our method has lower MAE and RMSE than other comparison models at all time steps, especially in long time step forecasting. This result shows that the hybrid model combining MFE and TCF has strong predictive ability and stability when processing complex time series data. Ablation experiments further verify the important contribution of each module to the overall performance. Removing any module results in a notable drop in model effectiveness, indicating that both modules are essential for capturing different dependencies in the data.

Our study not only demonstrates the potential of this hybrid model in network traffic forecasting but also provides new ideas and methods for its future application in other time series forecasting tasks. Future research can further verify the effectiveness of the model on larger and more diverse datasets, and explore combining other advanced technologies to further improve prediction performance.
